# Feasibility of repellent use in a context of increasing outdoor transmission: a qualitative study in rural Tanzania

**DOI:** 10.1186/1475-2875-13-347

**Published:** 2014-09-02

**Authors:** Onyango Sangoro, Ann H Kelly, Sarah Mtali, Sarah J Moore

**Affiliations:** Ifakara Health Institute, Box 74, Bagamoyo, Tanzania; Disease Control Department, London School of Hygiene and Tropical Medicine, Keppel Street, London, WC1E 7HT UK; Department of Health Interventions, Swiss Tropical and Public Health Institute, Socinstrasse, 57, CH-4002 Basel, Switzerland; University of Basel, Petersplatz 1, 4003 Basel, Switzerland; Department of Sociology, Philosophy and Anthropology, University of Exeter, Byrne House, Exeter, EX4 4PJ UK

**Keywords:** Repellent, Malaria, Knowledge, Attitude, Perceptions, Practice

## Abstract

**Background:**

Extensive employment of long-lasting insecticidal nets (LLINs) and indoor residual spraying (IRS) has substantially reduced malaria morbidity and mortality in sub-Saharan Africa. These tools target indoor resting and biting vectors, and may select for vectors that bite and rest outdoors. Thus, to significantly impact this residual malaria transmission outdoors, tools targeting outdoor transmission are required. Repellents, used for personal protection, offer one solution. However, the effectiveness of this method hinges upon its community acceptability. This study assessed the feasibility of using repellents as a malaria prevention tool in Mbingu village, Ulanga, Southern Tanzania.

**Methodology:**

Change in knowledge, attitude and practice (KAP) in relation to repellent use was assessed before and after the implementation of a cluster randomized clinical trial on topical repellents in rural Tanzania where repellent and placebo lotion were provided free of charge to 940 households for a period of 14 months between July 2009 and August 2010. Compliance, defined as the number of evenings that participants applied the recommended dose of repellent every month during the study period, was assessed using questionnaires, administered monthly during follow up of participants in the clinical trial. Focus group discussions (FGDs) were conducted in the same community three years later to assess the community’s KAP in relation to repellents and preference to different repellent formats.

**Results:**

At baseline, only 0.32% (n = 2) households in the intervention arm and no households in the control arm had ever used topical repellents. During follow-up surveys, significantly more households, 100% (n = 457) in intervention arm relative to the control, 84.03% (n = 379), (p = <0.001) perceived the repellent to be effective.

Post-study, 99.78% (n = 462) and 99.78% (n = 463), (p = 0.999) in the intervention and control arms respectively, were willing to continue repellent use. Mosquito nuisance motivated repellent use. From the FGDs, it emerged that most respondents preferred bed nets to repellents because of their longevity and cost effectiveness.

**Conclusion:**

High repellent acceptability indicates their feasibility for malaria control in this community. However, to improve the community’s uptake of repellents for use complimentary to LLINs for early evening and outdoor protection from mosquito bites, longer lasting and cheap formats are required.

**Electronic supplementary material:**

The online version of this article (doi:10.1186/1475-2875-13-347) contains supplementary material, which is available to authorized users.

## Background

Long-lasting insecticidal nets (LLINs) and indoor residual spraying (IRS) have had a great impact on malaria morbidity and mortality in the past decade in sub-Saharan Africa [1–3]. While effective, these tools are intra-domiciliary and predominantly target indoor biting and resting vectors [4]. This favours outdoor resting and biting vectors as IRS and LLINs are less effective against those vectors that exhibit exophily and exophagy [5]. Therefore, as malaria moves from sustained control to elimination, new tools that tackle residual outdoor malaria transmission are needed.

Repellents used outdoors and in the early evenings and mornings, where IRS and LLINs cannot be employed, present one strategy that can be used to push towards the goal of eradication. Topical (skin applied) repellents have been used as a form of personal protection for hundreds of years [6], and have been shown to protect against malaria in South America (80% reduction) [7] and Southern Asia (60% reduction) [8], and more recently in Ghana (34% reduction) [9] and Ethiopia (19% reduction) [10]. The major drawback to using topical repellents is compliance. Topical repellent use requires daily use and frequent re-application as their effects is usually short-lived over a few hours and therewith a change in daily routine (personal behaviour). While changing personal behaviour to use new interventions is not impossible as has been demonstrated in bed net campaigns [11], oral hygiene [12] and hand washing strategies [13], it is influenced by a number of other factors including: cost, perceived quality of the intervention, accessibility, information and ease of use. An intervention is likely to be used by the community if its affordable, perceived to be effective, the community is aware and has knowledge of its uses and finally, the intervention is simple to apply, i.e. it does not require considerable deviation from daily routine [14]. Therefore to influence behaviour change towards uptake of interventions: the community must be educated to improve information on the appropriate measures to employ to prevent disease e.g. use of bed nets to prevent mosquito bites and hence malaria infection. Secondly, the interventions must be made physically accessible to the community, such as considering the distance to shops where bed nets are sold or re-treated. Third, the cost of the intervention must be affordable and perceived as reasonable among community members to encourage use. Perception of the effectiveness of the intervention will also influence uptake, with the community more likely to use interventions they perceive as beneficial to them, for instance LLINs prevent mosquito bites. Lastly, is the ease of use of the intervention being implemented, as the community is more likely to use interventions that require the least deviation from daily routine, like use of drugs with simple dose regimens compared to those that have complicated regimens [14].

Therefore, in an effort to determine the feasibility of using repellents as a mosquito control tool, this study assessed the knowledge/awareness, acceptability, perceptions on effectiveness and preference to different kinds of repellents in a rural community in Kilombero valley, Southwest of Tanzania. The community in this setting has experienced extensive malaria research projects and intervention programmes spanning two decades [15–17] and was expected to be highly knowledgeable about malaria prevention and control. Cooking mainly takes place outdoors and in the early evening, a situation that exposes the community to nuisance mosquito bites and potential malaria transmission before they have employed bed nets. Further, like the rest of sub-Saharan Africa, the study area is experiencing rapid rural development, shifting the spaces and protocols of social behavior. Where once it was customary to retire shortly after sundown, now, owing to rural electrification programmes, residents usually gather in the early evening and stay late into the night at local bars and social centres springing up in the study area, thereby increasing perception of mosquito nuisance and malaria transmission potential at these times.

The dominant vector in this area is *Anopheles arabiensis*[18] that has been shown to shift to early evening and outdoor biting when hosts are unavailable late in the night indoors as a result of high bed net use [19, 20]. The presence of rice fields in the study area, as the community’s main occupation is farming, provides for a large breeding site of mosquitoes [21]. The presence of this large breeding site is likely increase mosquito abundance in the study area, and with it potential malaria transmission and nuisance biting.

Before the start of the clinical trial, the community were sensitised to the potential for repellents as a malaria prevention tool through skits, community meetings and leaflets. Therefore, they are likely to understand the importance of topical repellents in prevention of early evening malaria transmission potentially occurring in the study area before they go to sleep under bed nets, and are therefore more likely to be receptive to this intervention. Secondly, the customary practice of cooking outdoors as well as presence of electricity exposes this community to nuisance biting in the evenings as a result of the extensive rice fields present in the area, a situation likely to encourage use of repellent. Finally, repellents were provided free so the community were likely to use them and form an opinion on their efficacy.

## Methods

### Study area and population

This study was conducted in Mbingu village, Ulanga district, Tanzania, situated 55kms west of Ifakara town at 8.195°S and 36.259°E. There is malaria transmission all year round, with peak transmission occurring in the months of May and June after the long rains. The village experiences an annual rainfall of approximately 1,200-1,800 mm and an annual temperature range of between 20°C and 32.6°C. The village borders an extensive field cleared for irrigation, which provides an ideal breeding site for malaria vectors. The houses in the village are clustered in groups of 3–5 households, which mainly belong to one family, but in a few instances the houses may be rented by different families. In July 2009 (at the inception of the clinical trial), the population of the study area was estimated to be 7, 609, with each household having approximately 5 members [22]. Most houses are constructed from mud walls and thatched roof, with one‒third made from brick walls and corrugated iron roof.

### Outline of study

Between July 2009 and August 2010, a placebo-controlled cluster randomized clinical trial was conducted in the study village where 15% DEET (*N, N-*Diethyl-3-methylbenzamide) topical repellent and an identical placebo lotion were randomly issued to 940 households in the study village [23]. The clinical trial participants were also issued with double size LLINs per sleeping space to ensure equity. Treatments were issued to two study arms of 10 clusters with 47 households each. One study arm was issued with topical repellent lotion while the other study arm received a placebo lotion and both arms were followed up for 14 months to assess the malaria incidence between these two groups. Concurrent with the clinical trial, a knowledge, attitude and practice survey (KAP) of the repellents issued during the clinical trial was conducted by administering a questionnaire (Additional file 1: Repellent KAP survey tool) at the baseline of the clinical trial (before/entry survey) to assess community knowledge of repellents; at the beginning of every month when field workers visited the households to replace repellents that had run out (follow-up survey) throughout the study period, to assess the acceptance and compliance of the community to the repellent issued and perceived effectiveness; and at the end of the clinical trial (after/exit survey) to assess willingness to continue use of repellents. A separate Focus Groups study was conducted three years later in June 2013.

## Procedure

### Baseline survey

At baseline, written informed consent was sought from the household heads that were willing to participate in the clinical trial. The household heads gave consent for all household members who were below 18 years. Household members above 18 years were asked to sign their own written consent forms. As the household was analysed as a unit, a structured questionnaire of KAP in relation to repellents was administered to the household head. A unique ID was stapled on the door of each household that was recruited into the study.

### Follow-up survey

To assess acceptability and use, at the beginning of every month after the baseline survey, field workers visited the households recruited in the study to replace the tubes of repellent issued the previous month. A KAP questionnaire was administered during these visits, where the households were asked if they liked the repellent issued and their perceptions on the effectiveness of the repellent. The fieldworkers also administered a compliance questionnaire, where household members were asked if any household member had skipped a day of repellent use in the past month and reasons for missing that day. However, if during the follow up survey there were no household members present to answer the questionnaire on compliance, and continued to be absent for seven consecutive days after the first visit to assess compliance, that household was considered non-compliant to repellent use for that month. If the households reported that any household member did not use the repellent, that household member was removed from follow up time for the period they did not use the repellent. Thus, if all household members reported using repellent each night in the past week and an adult member of the household was present to be issued with new repellent, that household was considered compliant for the previous month. In addition, the number of treatment tubes (repellent and placebo tubes) issued per month was recorded, to determine if there was a difference in the number of tubes issued in each month per treatment group. Differences between recalled and observed compliance were not measured.

### Post-study survey

At the end of the clinical trial, (August 2010), an exit KAP (post-study) questionnaire to assess perceptions on effectiveness and willingness to pay if repellent was provided at cost was administered. In particular, the respondents were asked what was their perceived cost for the repellent issued during the clinical trial. The were also asked how much they were willing to pay for the tube of repellent they were given during the clinical trial.

## Focus group discussions

### In-depth discussions

Seeking an in-depth understanding of the knowledge, attitude, perceptions and practice in relation to repellents as a vector control intervention, a descriptive exploratory study, consisting of seven Focus-Group-Discussions (FGDs) and one Small Group Interview (SGI) was conducted in the study village from 10^th^ – 28^th^ June 2013, three years after the clinical trial. The participants may or may not have participated in the initial clinical study of topical repellents, as prior participation in the previous trial was not an inclusion or exclusion criterion. Several different formats of repellents were provided to participants to measure perceived preferences in delivery formats of repellents among members of a community that had previous familiarity with repellents.

### Sampling of FGD participants

This study initially used convenient sampling to enrol household heads in the village. A purposive sample of households with the following characteristic were drawn from the community:

- Households that had the males as household heads.
- Households that had females as household heads (widows, divorced, separated etc.).
- Households that had males as household heads but from which their female partners were invited for the FGDs and SGI.
- Households that had children of school going age (both primary and secondary schools).

From this sample, 6 – 12 individuals from households with each of the above characteristics were interviewed in seven FGDs and one 5-member SGI. The FGDs were dynamic in nature consisting of individuals from 10 to 60 years of age and sampling was stopped at the ‘point of saturation’ (no further ‘new’ information generated).

### Study tools

Based on literature on knowledge and practice in relation to repellent use and on *a priori* experience of repellent work with the community in the study area, an interview guide on perceptions and practices around repellent use in Mbingu village was developed for conducting the FGDs. This guide was pre-tested on four villagers, two men and two women before undergoing further changes based on the feedback from these villagers. The outcome was a simple interview guide that consisted of six open ended questions that were structured in a flexible manner to allow for any emerging ideas from the participants to be incorporated there in.

### Repellents explored

**Figure 1:**
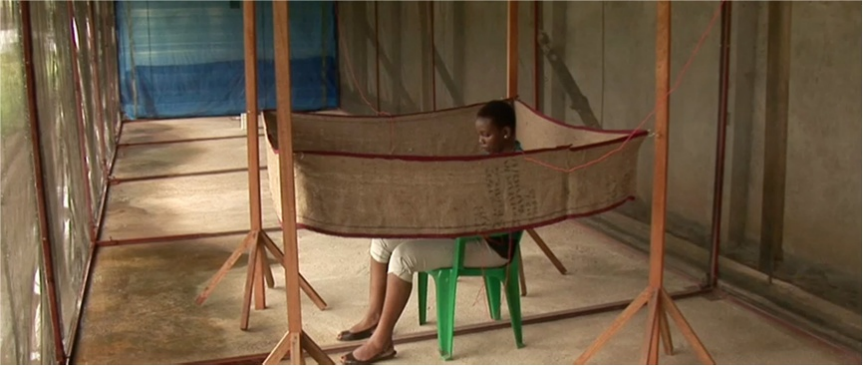
**Testing the efficacy of transfluthrin impregnated sisal strip in the semi-field system at the IHI.**

**Figure 2:**
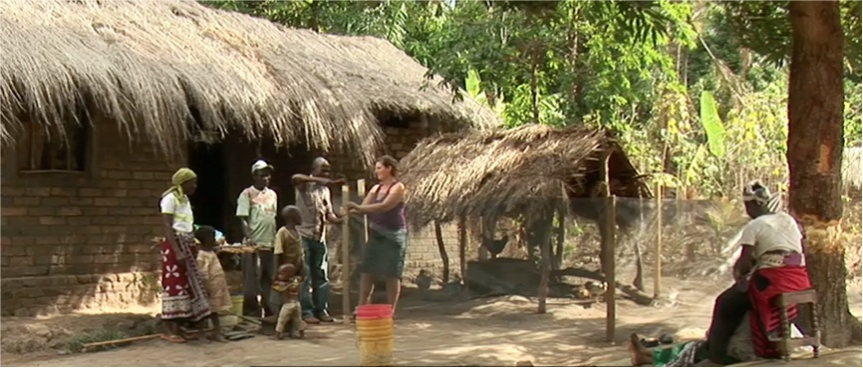
**Installation of permethrin impregnated fencing around an outdoor kitchen/cooking area in the study area.**

The different types of repellents issued to the participants of this study were; Permethrin impregnated ‘*kangas*’ (a sheet of fabric worn around the waist by women in Africa), 15% DEET (*N, N-*Diethyl-3-methylbenzamide) topical repellent in petroleum jelly format, 15% DEET topical repellent in spray format, 30% PMD (Para-Methane 3-8-diol) topical repellent in lotion format, 30% PMD topical repellent in spray format, 2% transfluthrin impregnated sisal strip (sack), that was hung in a common area where all household members sat, (Figure 1) [24] and 2% permethrin impregnated net fencing that was designed to protect individuals sitting outdoors, especially around the cooking area (Figure 2).

### Procedures

Participants were verbally informed on the objectives and aims of the study, its voluntary nature, risks and benefits. Thereafter verbal informed consent was sought from the purposive and final sample of participants after all ethical considerations of the study had been outlined. Interview schedules, including convenient interview times and venues were then negotiated between the study investigators and participants and the study commenced from the 10^th^ to 28^th^ of June 2013. The interviews were all conducted in Swahili and lasted between 30mins and 1 hour in the various local settings preferred by the participants. Consent was sought to use a tape recording device for the sessions with all villagers agreeing to be tape recorded prior to commencement of the interviews. First, four FGDs with the four different respondent groups: households that had the males as household heads, households that had females as household heads (widows, divorced, separated etc.), households that had males as households heads but from which their female partners were invited for interviews, and households that had children of school going age (both primary and secondary schools), were conducted where community knowledge (familiarity) and use of repellents as a mosquito control tool was assessed. At the end of these first four FGDs, the respondent groups were issued with different formats of repellents to use for a week. After using the different repellent formats for one week, these respondents groups were recalled for a further three FGDs and a single SGI where experiences of repellent use and preference to different repellent formats were assessed.

### Data management

Data from the baseline, follow-up, and post-study surveys were linked using the household unique identifier. Data from these questionnaires were entered into and coded using an Epi-info template that corresponded to the format of the questionnaires. All data was double entered into Epi-info, where it was checked for excesses or missing of data. Data was then exported to Microsoft Access 2010 database where it was checked for duplicates. Data from the FGDs was collected using tape recorders and imported into the computer where they were stored as audio files ready for transcription and analysis.

### Data analysis

All data analysis was carried out in STATA 11.2 (StataCorp LP, College Station, Texas, USA) software. Data from the baseline, follow-up and post-study surveys were analysed using descriptive statistics and are presented in tables (Tables 1, 2 and 3).

**Table 1:** **Baseline perceptions on malaria and repellents**

	Repellent n (%)	Placebo n (%)	Totals n (%)	P- value
***What is malaria***				
Disease	285 (93.44%)	270 (95.07%)	555 (94.23%)	0.397
Don’t know	20 (6.56%)	14 (4.93%)	34 (5.77%)	
***Causes of malaria***				
Mosquitoes	302 (99.01%)	280 (98.59%)	582 (98.81%)	0.634
Other	3 (0.99%)	4 (1.41%)	7 (1.19%)	
***Knowledge of malaria prevention methods***				0.664
Bed nets	286 (94.38%)	271 (95.42%)	557 (94.89%)	
Environmental management	7 (2.31%)	3 (1.05%)	10 (1.70%)	
Going to hospitals	4 (1.32%)	2 (0.70%)	6 (1.02%)	
Using repellents	1 (0.33%)	1 (0.35%)	2 (0.34%)	
Don’t know	5 (1.65%)	7 (2.46%)	12 (2.04%)	
***Knowledge of mosquito breeding site***				0.998
Water puddle	291 (95.40%)	270 (95.40%)	561 (95.41%)	
Other	14 (4.60%)	13 (4.60%)	27 (4.59%)	
***Protection methods used***				
Bed nets	294 (95.14%)	277 (96.85%)	571 (95.97%)	0.600
Mosquito coils	3 (0.97%)	3 (1.04%)	6 (1.01%)	
Environmental management	7 (2.26%)	5 (1.74%)	12 (2.02%)	
Covering oneself	4 (1.29%)	1 (0.34%)	6 (0.84%)	
Using repellents	1 (0.32%)	-	1 (0.17%)	
***Reasons for using protection methods***				
Effective	174 (56.31%)	154 (54.03%)	328 (55.22%)	0.008
Readily available	34 (11.00%)	22 (7.71%)	56 (9.34%)	
Cheap	23 (7.44%)	8 (2.80%)	31 (5.22%)	
Easy to use	76 (24.59%)	100 (35.08%)	176 (29.63%)	
Other	2 (0.64%)	1 (0.35%)	3 (0.51%)	
***Reasons for not using repellents***				
Don’t understand use	139 (45.27%)	118 (41.40%)	257 (43.41%)	0.057
Not aware of repellents	38 (12.37%)	28 (9.82%)	66 (11.15%)	
Not available	109 (35.50%)	115 (40.35%)	224 (37.84%)	
Expensive	16 (5.21%)	24 (8.42%)	40 (6.76%)	
Other	5 (1.62%)	-	5 (0.84%)	
***Willingness to use repellents***				
Yes	309 (99.67%)	286 (100%)	595 (99.83%)	0.336
No	1 (0.32%)	-	1 (0.17%)

**Table 2:** **Assessment follow up of households, repellent use and perceptions during the study period**

	Repellent n (%)	Placebo n (%)	Total proportions/treatment	P value
***Like repellent***				
Yes	462 (99.35%)	390 (84.41%)	852 (91.91%)	<0.0001
No	3 (0.65%)	72 (15.59%)	75 (8.09%)	
***Compliant***				
Yes	379 (81.50%)	361 (78.13%)	740 (79.83%)	0.202
No	86 (18.49%)	101 (21.86%)	187 (20.17%)	
***Perceived effectiveness***				
Yes	457 (100.00%)	379 (84.03%)	836 (92.07%)	<0.0001
No	0 (0.00%)	72 (15.96%)	72 (7.93%)

**Table 3:** **Assessment of perceptions on repellent use, effectiveness and cost after the study period**

	Repellent n (%)	Placebo n (%)	Total proportions/treatment	P- value
***Reasons for non-compliance***				
Forgot	35 (70.00%)	89 (60.13%)	124 (62.63%)	0.241
Away in the field	13 (26.00%)	56 (37.83%)	69 (34.85%)	
Don’t like repellent	1 (2.00%)	-	1 (0.51%)	
No mosquitoes	1 (2.00%)	2 (1.35%)	3 (1.52%)	
Ran out of repellent	-	-	-	
Other	-	1 (0.67%)	1 (0.51%)	
***Perceptions about repellents***				
Effective	455 (98.69%)	208 (45.61%)	663 (72.30%)	<0.0001
Easily available	5 (1.08%)	50 (10.96%)	55 (6.00%)	
Nice smell	-	99 (21.71%)	99 (10.80%)	
Smooth on skin	-	98 (21.49%)	98 (10.69%)	
Other	1 (0.21%)	1 (0.21%)	2 (0.22%)	
***Willingness to use repellent again***				
Yes	462 (99.78%)	463 (99.78%)	925 (99.78%)	0.999
No	1 (0.21%)	1 (0.21%)	2 (0.22%)	
***Willingness to pay***				
Yes	458 (99.78%)	455 (98.48%)	913 (99.13%)	0.034
No	1 (0.21%)	7 (1.51%)	8 (0.87%)	
***Perceived cost of repellent***				
< 0.6 USD	99 (21.80%)	111(26.74%)	210 (24.17%)	0.023
0.6 – 1.2 USD	280 (61.67%)	212 (51.08%)	492 (56.62%)	
1.2 – 1.8 USD	61 (13.43%)	75 (18.07%)	136 (15.65%)	
1.8 – 3.05 USD	13 (2.86%)	17 (4.09%)	30 (3.45%)	
> 3.05 USD	1(0.22%)	-	1 (0.12%)	
***Amount participants were willing to pay***				0.347
**<** 0.30 USD	388 (83.43%)	402 (87.77%)	790 (86.06%)	
0.30 – 0.60 USD	64 (13.91%)	52 (11.35%)	116 (12.64%)	
0.60 – 1.20 USD	7 (1.52%)	4 (0.87%)	11 (1.20%)	
1.20 – 1.52 USD	1 (0.21%)	-	1 (0.11%)	

Data from the socio-economic status (SES) was analysed using principal component analysis (PCA). A socio-economic index was generated using PCA and the generated score used to show wealth index of each household. Indicators of (SES) used were; asset ownership, household construction materials and education level of household head. These results are reported in detail elsewhere [23]. Data for KAP collected during the follow-up survey was analysed by determining trend over time, using descriptive statistics. Compliance data collected using the follow-up survey was also stratified by SES quintiles to determine if there was a difference in repellents use by SES quintile.

Data for KAP collected at baseline and post-study survey was analysed by comparing the before and after studies using descriptive statistics. Likewise, in the post-study survey, willingness to pay was compared across the SES quintiles.

The number of repellent and placebo tubes issued was analysed by linear regression against month, treatment group and an interaction of month and treatment group to determine if there was a significant difference in the number of tubes issued in each month and per treatment group.

Data collected over the study period (follow-up survey) was used to report outcomes on compliance, community liking the repellent and perception of effectiveness of repellents because it was assumed to be less prone to recall bias compared to data collected at the end of the clinical trial (post-study survey).

Audio files from FGDs were transcribed verbatim in Microsoft Office and imported into Nvivo 9 (QSR international Pty Ltd 2006–2010) qualitative analysis software. The data was the then coded into themes as they emerged from the response data in the transcripts. This content analysis also allowed for themes emerging from the data to be considered during iterative coding. The final coding tree (structure of categorizing data) consisted of identified themes from the data as well as unanticipated themes from the respondents. The final stage of the analysis involved re-organization of the themes into larger categories of themes communicating the key messages from each of the smaller themes under them (Table 4).

**Table 4:** **Major themes generated from the Focus group discussions (FGD’s) and Small group interviews (SGI)**

Major results theme	
**Theme 1**	Respondents were aware of the link between malaria and mosquitoes, but their knowledge on malaria aetiology and transmission was shallow. This did not however, effect their compliance with an intervention that was available free of charge.
**Theme 2**	Although respondents had adequate knowledge of repellents as a mosquito control tool, they preferred to use the bed net over repellents.
**Theme 3**	Those respondents aware of topical repellents had adequate knowledge on their proper use
**Theme 4**	Availability (access) and cost of repellents were major barriers to repellent use after the trial ended and repellents were no longer supplied.
**Theme 5**	The respondents perceived the repellents to be effective against mosquito bites, mostly in the early evenings.
**Theme 6**	Respondents recommended repellents be made more available and insecticides (permethrin) used to treat clothing be provided to enable self treatment.

### Ethical consideration

Participants were recruited on written informed consent. Ethical approval for the study was obtained from Ifakara Health Institute (IHI) (IHRDC IRB A46), Tanzanian National Institute of Medical Research (NIMR/HQ/R8a/VOL IX/780) and the London School of Hygiene and Tropical Medicine Ethical Review Board (LSHTM ERB 5174). IHI provided study monitoring.

## Results

### Baseline survey

**Figure 3:**
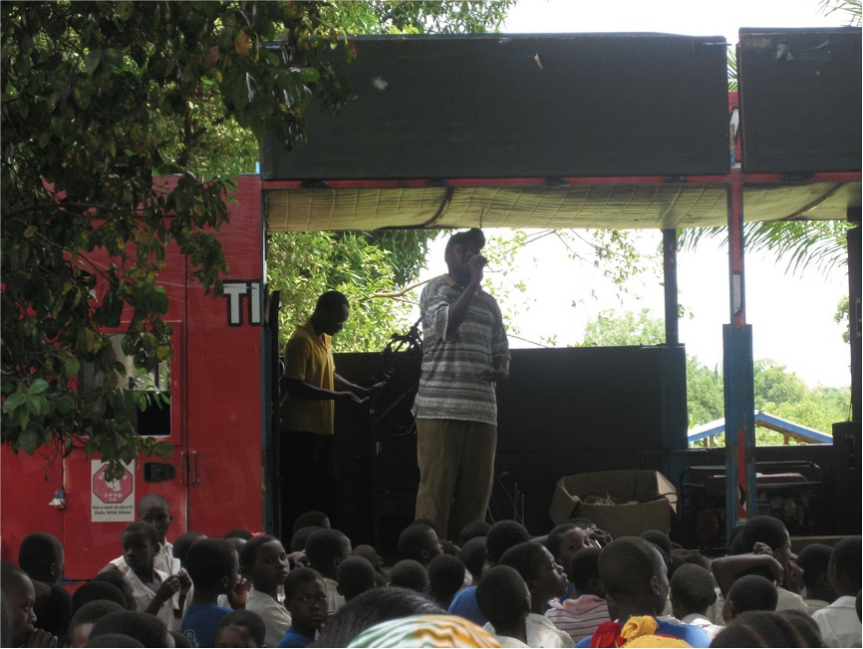
**Community sensitization meeting on repellents conducted by the social marketing team from IHI.**

At baseline, only 0.32% of the households had ever used repellents in the intervention arm, while no households had ever used repellents in the control arm (Table 1). Two households reported burning mosquito coils, five households repelled mosquitoes with a smoky fire and one household reported using repellent plants (data not shown). Most households (95.7%) used bed nets as these had been delivered through various governmental and non-governmental schemes from 1997 onwards. When asked about malaria a similar proportion of the households in the intervention and control arms reported that malaria is a disease: 93.44% (n = 285) and 95.50% (n = 284), respectively. When asked about malaria transmission, most households in the intervention arm 99.01% (n = 302) and control arm 98.59% (n = 280) reported that mosquitoes transmit malaria. Bed nets were the major prevention tool used in the study village, with a similar proportion of reported bed net use in the intervention 95.14% (n = 294) and control arm 96.85% (n = 277). When households that reported bed net use, were further asked why the preferred bed nets to other tools, a significantly larger proportion cited effectiveness relative to other reasons: 56.31% (n = 174) and 54.03% (n = 154) in the intervention and control arm, respectively. Other reasons for use of bed nets as well as other mosquito bite protection methods are reported in Table 1. It should be noted that the bed nets reported by the respondents, were not those issued during the clinical trial, but they were reporting on tools they used before the onset of the clinical trial. However, bed nets were given at the start of the clinical trial to ensure equity between the study arms. An equal proportion of households in both the intervention 95.40% (n = 291) and control 95.40% (n = 270), arms reported that mosquitoes breed in standing water. The major barrier to repellent use in this community was lack of knowledge on how to use repellents, with 45.27% (n = 139) households in the intervention and 41.40% (n = 118), in the control arm reporting that they did not understand how topical repellents were used. Lack of awareness of repellents was also reported as a barrier to repellent use, with 35.50% (n = 109) and 40.35% (n = 115) of the households in the intervention and control arms respectively, indicating that they were not aware of repellents as a mosquito control tool. However, when repellents were made available knowledge was no longer a barrier to compliance. All households were willing to use repellents to prevent mosquito bites: 99.67% (n = 309) of the households in the intervention and 100% (n = 286), (p = 0.336), in the control arm were willing to use repellents, even though this tool was novel in this community after community sensitization, (Figure 3).

### Follow-up survey

A follow up survey was conducted to assess household compliance to repellent use. Compliance in this context is defined as having recalled use of the repellent every night in the past month. However, if during the follow up survey there were no household members present to answer the questionnaire on compliance, and continued to be absent for seven consecutive days after the first visit to assess compliance, that household was considered non-compliant to repellent use for that month. If the households reported that any household member did not use the repellent, the household member was removed from follow up time for the period they did not use the repellent. Reported household compliance with repellent use was not significantly different between the study arms: 81.50% (n = 379) in the intervention and 78.13% (n = 361) in the control arm, (p = 0.202) during the study period. Significantly more households liked using the repellent in the intervention arm 99.35% (n = 462) compared to the control arm, 84.41% (n = 390), (p = <0.0001). When asked about effectiveness, significantly more households in the intervention arm, 100% (n = 465) compared to the control arm 84.03% (n = 379), (p = <0.0001), perceived repellents to be effective (Table 2). Also, significantly more households that perceived the repellent to be effective complied with repellent use (72.31%) compared to those households that did not comply (27.68%), (p = <0.0001). This indicates that relief from mosquito bites was a motivating factor in repellent compliance.

When the perceptions of effectiveness of repellents was analysed over the study period, it was observed that there was an increase in the number of households reporting the repellent to be effective over time. This trend was also observed for households that reported to like the repellents. Compliance was observed to increase over the study period, with more households reporting repellent use at the end of the study compared to the start of the study. Because the repellents were given out for free there was no difference in repellent compliance between the most poor and least poor socioeconomic quintiles (p = 0.369), data not shown.

There average number of tubes issued per household was 6.73 (95% C.I. 6.51 – 6.95) and 6.92 (95% C.I 6.68 – 7.16) per household per month in the intervention and control groups, respectively and there was no significant difference between the treatment arms: Odds Ratio 1.68 (95% C.I. 0.32 – 84.25, P = 0.803) and this remained constant for the duration of the study period.

### Post-study survey

The main reason for non-compliance to interventions was forgetfulness, with 70% (n = 35) of the households in the intervention and 60% (n = 89), (p = 0.241) in the control arm reporting that the major reason they did not comply with the intervention at some point during the study was because they forgot to apply the repellent. Travel also lead to non-compliance with 26% of households in the intervention arm and 37.83% of households in the control arm not complying for a month because they had gone to work in the fields.

When asked why they liked using the repellents, significantly more households in the intervention arm 98.69% (n = 455) relative to the control arm 45.56% (n = 208) cited effectiveness, (p = <0.0001). It is worth noting that all households who mentioned nice smell and smooth feeling on the skin as reasons for using repellents were from the placebo arm of the trial. When asked if anyone in their household suffered from malaria during the trial, significantly more participants from the placebo arm answered yes: 32.9% versus 15.5%, (p < 0.0001).

Equal proportions of households were willing to continue using repellents after the clinical trial (Table 3). When asked if they would be willing to pay if the repellent was made available at a fee, 99.78% (n = 458) of the households in the intervention and 98.48% (n = 455), (p = 0.999), in the control arm reported that they were ready to pay a small fee, with majority of the households in the intervention, 84.34% (n = 388) and control arms 87.77% (n = 402), (p = 0.347) willing to pay at most $ 0.30 for a tube of repellent (Table 3), even though all participants perceived that the value of the repellent was at least double that figure. There was no difference in willingness to pay when SES quintiles were compared (p = 0.668).

### Focus group discussions

#### Perceptions around malaria control and transmission

To provide a general picture of the community’s knowledge, attitude and practice in relation to malaria and ways to control malaria, participants were questioned about their knowledge of malaria transmission and methods of prevention and control used. Some of the participants had a comprehensive understanding of malaria and control, as observed from the response of one female respondent below: “*Malaria is caused by a female mosquito when it bites you at midnight”* (Meeting group 5, 16^th^ June 2013).

Interestingly however, and especially in a region where there has been consistent malaria control, research and intervention implementation by both non-governmental and governmental organizations for over 20 years [15–17], the community members did not appear to have an in depth knowledge of malaria transmission. In trying to assess the depth of community knowledge on the malaria transmission process, the respondents were asked how many times a mosquito had to bite a person for it to transmit malaria. Most of the respondents did not seem to know:

“*We do not know unless you tell us”*- (Meeting group 4, 14^th^ June 2013).

“*Many times*”- (Meeting group 1, 14^th^ June 2013).

This indicates the community knowledge on malaria transmission is superficial, so that whilst the community are aware that mosquitoes transmit malaria, their knowledge on this transmission process is scant. These gaps in knowledge might suggest a bias during implementation of malaria control programmes, so that, rather than promoting community sensitization and education on the objectives of the intervention, the link between intervention and disease, and the benefits of the intervention to the individual and the community, these programmes likely focus more on coverage of the control tools.

#### Preference of malaria prevention tools used in the community

All respondents had used some form of personal protection against mosquito bites even for those who weren’t quite sure what malaria was. It also emerged that they had been using these tools for a long time and were convinced that the tool each one of them had been using was the most effective. The most commonly reported malaria prevention tool used was the bed net, when respondents were asked which tool they used to protect themselves from mosquitoes and malaria:

“*We use nets*” – (Meeting group 1, 14^th^ June 2013).

Even though some of the respondents were aware of mosquito repellents and/or had acquired topical and spatial repellents at some point in the past 2 years, during or after the clinical trial, most of them still preferred using the bed net;

“*I would prefer the net*” – (Meeting group 3; 25^th^ June 2013)

When the respondents were questioned on why they preferred the bed net to other mosquito control tools, two major reasons were given. The first was cost effectiveness:

“*Because mosquitoes will not bite you when you are sleeping under a net but for the repellents they last for a short time and when the smell wears off then the mosquitoes bite you*” – (Meeting group 2, 26^th^ June 2013).

The second was generally the ease of use:

“*MG: FR: 03: Because it is not cumbersome*”- (Meeting group 3, 25^th^ June 2013).

#### Familiarity of topical repellents

At the onset of the FGDs, most respondents’ awareness of repellents was thin, with almost half of them largely unaware of topical repellents as a malaria control tool. However, those who had heard of topical repellents had adequate knowledge on the proper technique of using/applying the repellents as illustrated by the following quotes when respondents were asked how repellents were used;

“*You can apply and then it stays for a few hours after that it is no longer effective and the mosquitoes can bite you. After you apply it you have to wash your hands well with soap*” – (Meeting group 5, 16^th^ June 2013).

For those who knew about repellents, the primary source of information was outreach from the Ifakara Health Institute (IHI), previously Swiss Tropical Institute Field Laboratory (STIFL), which is the institute under which the clinical trial project was conducted. When asked how they came to know of repellents most respondents mentioned the clinical trial, which distributed the repellents free of charge:

“*They were being distributed by people from STIFL (IHI)*” – (Meeting group 1, 14^th^ June 2013).

#### Reported experience of use after topical repellent distribution and use

After repellent distribution, all respondents reported that they had used the repellent intervention issued to them during the second phase of FGDs. The most commonly reported reasons for continued use of the repellents by the respondents were mainly because of their effectiveness against mosquito bites and also because of the appeal in odour and presentation:

“*I liked it because it prevented mosquitoes and its smell did not affect us in any way like causing flu or any other effects*”- (Meeting group 3, 20^th^ June 2013).

Another reason that emerged from the interviews was that every member of the household could use the repellent as opposed to other interventions issued which only a few household members used:

“*I would choose the applying repellent because it can be used by the children, my husband and even visitors*”- (Meeting group 3, 20^th^ June 2013).

There was one report of side effects to repellent use, however this was during the clinical trial and not in the FGD study:

“*Yes I know my sibling he used to get rashes all over the body so he was told not to apply the repellents anymore*”- (Meeting group 5, 16^th^ June 2013).

#### Preference for different applications of repellents

After exposing the respondents to typical topical repellents containing active ingredients such as DEET and PMD and in various formats such as lotion, jelly, spray, permethrin impregnated clothing, (*kanga*), transfluthrin impregnated sack cloth and permethrin impregnated net fencing, the respondents expressed the following views and preferences;

“*I found the smell to be too strong*” when asked about DEET in spray format –(Meeting group 2, 25^th^ June 2013).

“*I liked the smell* “ when asked about PMD in lotion and spray formats – (Meeting group 2, 25^th^ June 2013).

“*I did not like the smell because it was too strong* “ when asked about DEET in jelly format – (Meeting group 2, 25^th^ June 2013).

“*The applying repellent because everyone can use it but the kangas cannot be used by everyone*” when asked to choose between topical repellents and insecticide treated clothing ‘*kanga’* – (Meeting group 3, 20^th^ June 2013).

“*If you sat near the sack repellent then the mosquitoes couldn’t bite you but if you sat just a distance away then they would bite*”– when asked about the transfluthrin impregnated sack, (Meeting group 6, 20^th^ June 2013).

“*I got the net so I used to sit inside it and the mosquitoes were very few. They used to bite the feet only but I could stay for like half an hour without bothering with any mosquitoes*” – when asked about the permethrin impregnated net fencing, (Meeting group 6, 20^th^ June 2013).

#### Factors that determine the continued use of topical repellents

For those who did not use repellents during the clinical trial, repellents were generally not popular. There were a several barriers to repellent use in this community; the first being access to the repellents:

“*We were given repellents for applying but after they got finished I have not used anything else apart from nets*” – (Meeting group 1, 16^th^ June 2013).

Repellents were provided of free during the clinical trial. However after the clinical trial, the community was unable to access repellents as they were not available in shops and drugs stores in the study area, as was highlighted by the respondent above.

The costs of the repellents according to most respondents limited their affordability with most respondents prioritizing other living essentials over the repellents. When asked to choose between buying a soda or the repellent (subject to availability), most of the respondents opted to buy the refreshment:

“*I would buy the refreshment or a net otherwise I would just use a lot of clothing to cover myself* ” – (Meeting group 1, 16^th^ June 2013).

#### Community recommendations on improving repellent use

In an effort to understand how to improve the use of repellents, participants were questioned on what they felt was necessary to make the interventions better. While most of the responses revealed that the repellent application was fine the way it was, other recommendations included the cost of the repellent:

“*I wouldn’t buy them because that is expensive unless you sold them in 500 shillings bottles* ” – (Meeting group 1, 16^th^ June 2013).

It should be noted that the bottle the respondent was recommending to be sold for 500 TZS/$0.30 contained 120 ml of repellent.

Odour of DEET repellent:

“*I did not like the smell because it was too strong*” – (Meeting group 2: Male respondent).

Issuing extra insecticides so that they could re-treat the impregnated clothing issued:

“*I also think that you should give us repellents for the kangas so that we can treat them once we wash them*” – (Meeting group 3, 25^th^ June 2013).

## Discussion

Despite the proven efficacy and acceptability of repellents for prevention of malaria [7–10], knowledge and utilization of repellents as a malaria control tool is low in sub-Saharan Africa. Lack of awareness of repellents as a malaria control tool is one of the major barriers to repellent use in sub-Saharan Africa. As observed from the baseline survey at the start of this study, most respondents had not used repellents before the implementation of the clinical trial. Therefore, use of topical repellents was completely new in this community as similar to several other studies conducted in the African continent [25–27]. It is evident that improving community knowledge and awareness [26–29] as well as retooling interventions to community needs and preferences [30] will improve the acceptability and uptake of interventions being advocated. The most commonly used malaria control tool in the study area was bed net. Social marketing of LLINs started in Kilombero and Ulanga district in July 1997, under the KINET project. At the launch of this programme the community was educated on malaria transmission and control [15]. This campaign was followed by the launch of the Tanzania National Voucher System (TNVS), implemented by the National Insecticide Treated Nets programme (NATNET), under the National Malaria Control Programme (NMCP) of Tanzania, from 2004. In 2007, the Ministry of Health and Social Welfare (MoHSW), collaborating with other partners launched the under five Catch-up Campaign, parallel to the TNVS programme. In 2008, the MoHSW and partners launched the Universal Coverage Campaign (UCC) [31]. Therefore, if repellents and indeed any other novel tools are to be accepted and used to complement LLINs and IRS, there will be a need for social marketing, community education and sensitization to be employed for a substantial period of time. It is also essential to determine community preferences. Tools that require daily compliance are initially likely to have limited uptake, as the community has to remember to adhere to them on a daily basis. As observed from the FGDs study, ease of use was one of the reasons why the community preferred bed nets to repellents. This was because, once hung, the bed net was used over a long period of time as you simply pull it down when you get into bed, compared to having to remember to apply the repellent every evening. However, ease of use was not the only factor that effected compliance. In the follow-up surveys, it was observed that there was lower compliance in the control arm relative to the intervention arm. Likewise, in the after study, it was observed that more households in the intervention arm relative to the control arm used the repellent because it was effective. This finding demonstrates that compliance to interventions does not only depend on its availability and ease of use but also on its effectiveness.

In the FGDs it was also observed that even though sisal impregnated sisal strips did not require daily compliance and were easy to use, they were reported to be effective over very short distances, and this discouraged the community from using it.

These finding demonstrates that to impact compliance, the efficacy of the tools being recommended need to be established. A recent mathematical model demonstrated that the effectiveness of any repellent is extremely dependent on two factors: efficacy and compliance (Moore and Briet, in preparation). The most effective tools are those that have high efficacy and require little user compliance such as house screening [32].

The major reason for use of topical repellents by the community in Mbingu village is to prevent nuisance biting by mosquitoes. Although a proportion of the community could associate mosquito bites with malaria, the results of this study imply that they used repellents to avoid being bitten by mosquitoes rather than to avoid contracting malaria. These results were similar to a study carried out in a coastal community in Mexico, where 80% of the respondents said they allowed IRS in their households to reduce mosquito bites while only 2% said they allowed IRS to avoid contracting malaria [33]; and in rural Tanzania, where respondents reported that main reason for using LLINs was to prevent mosquito bites: 73% of the respondents reported they allowed IRS in their households to reduce mosquito bites and only 17% related protection from mosquito bites with reduction of malaria in the family [25]. These findings demonstrate that tools being advocated as interventions, especially in malaria control should address both short and long-term goals, i.e. address the problem of nuisance biting or mosquito densities (efficacious to enhance uptake) as well as reduce disease prevalence/incidence in the long run (resultant effectiveness). This is likely to encourage uptake and acceptability as opposed to tools whose benefits are realized in the long-term, and highlights the need to test new vector control interventions against nuisance biting insects as well as target vectors during development for a better understanding of how effective that tool will be in the real world for disease control purposes.

The major reason for not using repellents in this community was reported to be lack of knowledge of repellent use and is similar to findings in other studies [26], where low repellents use was associated with poor knowledge of repellents. Availability of repellents in this community was another barrier to repellents use as observed from the baseline survey.

Also, in the FGDs, after the clinical trial, when asked why they did not use repellents, the respondents cited availability as a barrier, reporting that they did not know where to access repellents. Observations carried out by the study investigators during the clinical trail and FGDs, indicated that no topical repellents were available in the shops and drug stores the study area. Therefore, despite most households indicating willingness to continue repellent use, and even pay a small fee, access to repellent was a major barrier to repellent use.

Another barrier to the use repellents was cost [34]. During the FGDs, even though all respondents were aware of repellents as a mosquito control tool, they all preferred using LLINs as they reported that repellents were more expensive in the long run because they had to be replaced every end of the month compared to LLINs, which could last up to five years before replacement, if well taken care of. This finding was consistent with outcomes from other KAP studies assessing uptake of interventions [25, 28, 35]. As seen from the above studies, cost of mosquito control interventions greatly influences the acceptability and uptake in communities where they are to be employed. In rural and urban areas in Tanzania, a 150 ml bottle of 15% DEET repellent costs USD $1.00. On average, respondents were willing to pay $0.32 for a 150 ml tube of repellent that would last one adult less than one month. The current price of repellents is too expensive for the subsistence farmer, who lives on $1.50 USD per day. Therefore, even though incorporation of repellents into malaria control programmes on a community scale, is likely to use a cheaper but efficacious option of repellent, as was the case in Ghana [10], it is unlikely that the repellents would be subsidized down to or lower than $0.32. Also, scale up and extensive use of repellents under programmatic conditions as well as emergence of a repellent market is bound to drive the cost of repellents down. However these cost are unlikely to be lower than the cost of delivering a single LLIN, which costs USD $5.30 and protects two people for up to 5 years ($0.50 per person per year) [31]. Therefore if we are to encourage up take of repellents as a malaria control tool, the cost needs to be greatly reduced, potentially through government and non-governmental organizations offering subsidies on repellents following the example of LLINs [23]. The government may also encourage local production of repellents through tax exemption for local repellent manufacturers.

From the FGDs, it was observed that knowledge on malaria transmission and control was relatively superficial. While most respondents associated mosquitoes with malaria, when probed, few were able to detail processes of transmission, aetiology and prevention in any depth. Therefore, although all respondents from the FGDs reported that they used the repellents issued, it is likely that they did so with only a superficial understanding of the objectives of using repellents. This might have been because the community were more concerned with preventing mosquito bites than contracting malaria, as observed in other studies [25, 33]. As all respondents reported that they had complied with repellent issued it was not possible to assess the relationship between compliance and level of knowledge of malaria transmission. The superficial knowledge of malaria transmission observed in this community underscores the importance of incorporating community education and sensitization before implementation of any intervention to achieve its desired objective. Social marketing the product, and neglecting key messages regarding how these interventions benefit the communities in which they are being implemented, is likely to negatively effect uptake of that intervention. It is therefore essential for the community to be involved in designing and implementation of intervention programmes so that they have a better understanding of the objectives and use of tools being employed.

Several studies have shown that there has been better uptake of interventions in communities where awareness and sensitization have been conducted [36]. Promoting knowledge and awareness also deters any misconceptions that the community may have towards a particular intervention and it is essential for effective implementation of that intervention [37, 38]. During FGDs for this study, some respondents reported that they had ‘heard’ that LLINs caused infertility and also claimed that if/when they use repellents then their skin pores will be blocked and they will get sick. However in a KAP study in Rukungiri, Uganda, women who had previous knowledge of the use of ivermectin were more involved in making decisions of how ivermectin should be distributed to the community compared to those women who had no prior knowledge of this drug [39]. It is therefore essential to acknowledge and address the community’s misconceptions and misinformation about intended interventions in order to improve acceptability, uptake and effectiveness. Rather than the implementing organizations solely marketing the product to achieve extensive coverage, it is beneficial to also educate the community on the safe use of these interventions and the correlations between their products, the disease and its benefits.

The respondents’ preference of LLINs to repellents is attributable to cost effectiveness, convenience of use and availability. The major reason given for non-compliance to repellent use was that the respondents ‘forgot’ to use it, while ease of use was ranked second among reasons why respondents preferred using bed nets. It was cumbersome to remember to re-apply the repellent after every few hours, unlike simply sleeping under a LLIN. Repellents should therefore be presented in a format that will encourage uptake. As the major occupation in the study area is subsistence farming, most community members bathe in the evening after coming from their farms. Repellents can be incorporated into body lotions so that they are applied after taking the evening bathe. Repellents can also be impregnated in clothing, especially in *kangas* used by women in the evening when cooking outdoors. Development of tools that do not require daily compliance such as long lasting spatial repellents that act over long distance should also be explored [40].

Respondents also preferred LLINs because it protected them when they were asleep and vulnerable to mosquito bites as opposed to when they were awake and could chase mosquitoes away. The community however preferred to use repellents in the early evenings when sitting outside their houses to have a chat with other family members and friends without being bothered by mosquito bites. This finding is important because it suggests that repellents can be used complimentary to LLINs in the early evening, before LLINs are employed, which was a major objective of this study.

Perceived irritating odour of DEET topical repellents reduced its use by the community in this study, a finding similar to studies in North Tanzania and Mexico where participants refused to use IRS because of the ‘bad smell’ of insecticides used [25, 33], emphasizing that interventions should be tailored to be perceived as pleasing by users. PMD was perceived as pleasant as found in several other studies [7, 10, 41].

The most salient recommendation that came out of this study was that interventions advocated to the community should fit the community needs, such as providing repellents that have a pleasant smell and feels good when applied to the skin. Respondents that were issued with Permethrin impregnated *kangas*, reported that even though effective, it only protected a single individual at a time and suggested that all members in the household be issued with a treated *kangas*, and like LLINs, be issued with the ‘chemicals’ (insecticides) used for re-treatments so that when the effect of the insecticide was diminished they could treat the clothing on their own. Insecticide Treated Clothing (ITC), has been successfully implemented in other settings [42–45] and therefore this tool would easily be introduced in this community. Another outcome of this study was the effectiveness of the topical repellents that were issued. The respondents found topical repellents to be effective in protecting against early evening biting outdoors. This finding is similar to other studies, where repellents have been used to protect against vectors biting outdoors and by extension reduce the incidence of malaria [7, 8, 46]. Therefore both topical repellents and ITC, if designed to meet the needs and preferences of the community, could offer potential interventions that could be introduced for malaria control and would be readily accepted by the community.

## Conclusion

In this setting, the major limitations to use of repellents, similar to those identified from other studies were lack of knowledge, availability of repellent, cost and need to remember to use it every evening or even more than once in a single evening. While the community was highly knowledgeable about malaria, their knowledge was found to be superficial, indicating poor community education and sensitization. Although currently LLINs are the most commonly used and preferred malaria prevention and control tool, their introduction to the community was initially marked by similar limitations emerging from this study such as the need to use it daily and the cost being prohibitive. When repellents were provided free of charge to all trial participants compliance was high. It is therefore likely that uptake will improve if accessibility of repellents is improved through lower costs and greater availability through the commercial sector, comprehensive social marketing and community sensitization on use of repellents, as well as delivery of repellents in formats that respond to community desires. Even though LLINs were the preferred mosquito protection tool, the community saw a benefit in the use of topical repellents in the early evening, especially to prevent mosquito nuisance indicating the potential of using repellents complimentary to LLINs. However, longer lasting repellents are an essential requirement to avoid the need for frequent reapplication that most people find off-putting. The difference in compliance reported during and after the study is likely due to recall bias at the end of the study. Other avenues such as long lasting spatial repellents might be used if they are effective enough to protect the peridomestic space occupied by the family and visitors in the evening.

### Limitations to the study

A ranking of repellent preference had previously been reported in this study, but as there were too few repellents types/formats to issue to each FGD participant, these results were discarded along with some themes that had earlier been reported as they did not represent true results of community preference to different repellent formats.

As the participants were only issued with one repellent, it was not possible to explore whether the participants would use the repellents complimentary to each other if they had been issued with different formats of repellents. However, findings from the FGDs indicated the community members used the tools complimentarily.

Another limitation of this study is that compliance, during the follow up and post-study surveys, was established by self-reporting of use by the study participants. It was not logistically possible to observe compliance of households to repellents use for each household every evening and therefore observed and reported compliance could not be compared, and this should be taken into consideration when interpreting the results of this study.

## Electronic supplementary material

Additional file 1: **Repellent KAP survey tool.** (PDF 173 KB)
