# Supplementary material for: Feasibility of repellent use in a context of increasing outdoor transmission: a qualitative study in rural Tanzania
Source: Malar J. 2014 Sep 2;13:347. doi: 10.1186/1475-2875-13-347 (PMC4283126; doi:10.1186/1475-2875-13-347)
Supplement: Supplementary file 1 — Additional file 1: Repellent KAP survey tool. (PDF 173 KB) [file 12936_2014_3629_MOESM1_ESM.pdf]

IFAKARA HEALTH INSTITUTE
research | training | services

Community Perception of Repellents

Q1. Je wewe au mmojawapo kwenye kaya yako ameugua malaria kwa mwezi moja uliopita? Have you or anyone in your household suffered from malaria in the past one month?
1= Ndiyo Yes **2=Hapana** No **3= Sijui** Don't Know | _ |

Q2.Malaria ni nini? What is malaria?

___

___

Q3. Unafikiri malaria unasababishwa na nini? *(Usimsomehe)* What do you think causes malaria? *(Do not read out the answers)*

1= Ndiyo Yes **2=Hapana** No **3= Sijui** Don't Know

1. **Mbu** Mosquito | _ |
2. **Majira ya baridi au mvua** Cold weather | _ |
3. **Mafua** Flu | _ |
4. **Maji machafu** Dirty water | _ |
5. **Vinginevyo** Other____

Q4. Je unajua jinsi ya kuzuia kupata malaria? Do you know how to prevent contracting malaria?

___

___

___

Q5. Ni maeneo gani mbu huzaliana? *(Usimsomehe)* Where do mosquitoes breed? *(Do not read out the answers)*

1= Ndiyo Yes **2=Hapana** No **3= Sijui** Don't Know

1. **Maji yaloyotuama** stagnant water | _ |
2. **Mashamban**i Fields | _ |
3. **Mitoni** Rivers | _ |
4. **Kwenye vyoo** Pit latrines | _ |
5. **Kwenye takataka** Dirty environment | _ |
6. **Kwenye udongo** Soil | _ |
7. **Vinginevyo** Other____

Q6. i) Je, wewe na wengine kwenye Kaya yako mnafanya nini ili kujizuia kuumwa na mbu? What do you or your household normally do to prevent yourselves from getting mosquito bites?

Q6. ii) Kwa nini munapendelea njia hii? Why do you prefer this method?

**Njia**(Method)	**Ndiyo** (Yes)**=1** **Hapana** No**=2**	**Bei Nafuu**(Cheap)	**Inapatikana kwa urahisi** (Readily available)	**Rahisi kutumia** (Easy to use)	**Ina uwezo zaidi**(Effective)	**Sababu Nyingine**(Other) *Andika(Write)*
**Kujifunika (**Covering yourself)						
**Kufukiza moshi (**Smoky fire)						
**Dawa za kupaka mwilini (**Repellents on the body)						
**Kupuliza dawa za kuua ukutani (**Using insecticides)						
**Kuchoma dawa za dukani** (Burning mosquito coils)						
**Kujaza mashimo yaliyo na maji ya kusimama** (Fill puddles)						
**Kufyeka maajani** (Clear away bushes)						
**Kutumia vyandalua** (Use bed nets)						

Q7.Je wewe na wengine kwenye kaya yako mnatumia dawa za kijipaka mwilini ili kuzuia kuumwa na mbu?Do you or anyone in your household use mosquito repellents? ***(Kama hapana ruka hadi swali la Q.17)*** *(If no go to Q.17)*
|_|

1= Ndiyo Yes **2=Hapana** No **3= Sijui** Don't Know

Q8. Dawa hiyo inaitwaje? What is the name the repellent? ____

Q9. Dawa hiyo inapatikana wapi? Where do you get the repellent from? |_|

1. **Duka la dawa** Pharmacy
2. **Dukani** Shop
3. **Zahanati** Dispensary
4. **Kwinginepo** Other ____

Q10.Dawa hiyo ni ya aina gani? What type of mosquito body repellent do you use? | _ |

1. **Ya kupuliza** Spray
2. **Losheni** Lotion
3. **Mafuta ya kupaka** Oil
4. **Jeli** Jelly
5. **Nyinginezo** Other____

Q11. Dawa hiyo inajazwa kwenye nini? In what is the repellent packaged?

1. **Chupa** Bottle
2. **Mkebe** Tin
3. **Tyubu** Tube
4. **Plastiki** Plastic
5. **Nyinginezo** Other____

Q12. Dawa hizo zinapakwa wapi? Where do you apply the repellant? | _ |

1. **Mikono na miguu peke yake** (Limbs only)
2. **Mwili mzima** (Whole body)
3. **Sehemu za mwili pasipo na nguo** (Exposed areas only)
4. **Sehemu nyingine** (Other)____

Q13. Ni kitu gani kinachokupendeza kwenye dawa hizo za kufukuza mbu? What do you like about the repellant?

1= Ndiyo Yes　　**2=Hapana** No　　**3= Sijui** Don't Know

1. **Inanukia** (Smells nice**)** | _ |
2. **Ni nzuri kupaka** (Feels good on the skin) | _ |
3. **Ina rangi inayopendeza (**Has a nice colour) | _ |
4. **Inapatikana kiurahisi** (Readily available) | _ |
5. **Bei nafuu** (Cheap) | _ |
6. **inazuia kuumwa na mbu** (Protects against mosquito bites) | _|
7. **Nyingine** (Other) ____

Q14.Unaupaka dawa hizo mara ngapi kwa mchana au usiku? How many times do you apply the repellent in a day/night? | _ |

Q15. Unapaka dawa wakati gani? When do you apply the repellent? | _ |

1. **Machweo** (At sunset)
2. **Kabla ya chakula cha jioni** (Before supper)
3. **Baada ya chakula cha jioni** (After supper)
4. **Kabla ya kulala** (Before bed)
5. **Nyingine (**Other) ______________________________________

Q16. Kwa kawaida ni wakati gani unaoingia ndani ya nyumba wakati wa jioni? What time do you go into the house in the evening? | _ | _ |

Q17. Kwa kawaida unaenda kulala wakati gani? What time do you go to bed? | _ | _ |

Q18.Sababu zipi zinakufanya usitumie dawa za kupakaa mwilini ili kuzuia kuumwa na mbu? | _ |
Why don't you use mosquito body repellents?

1. **Hatujasikia** (I have never heard of them)
2. **Gharama** (Expensive)
3. **Haipatikani** (Not readily available)
4. **Hatuielewi** (Don't understand)
5. **9. Nyinginezo** (Other) ____

Q19. Je kama masuala ya hapo juu yakipatiwa ufumbuzi mko tayari kujaribu kutumia dawa za kupaka mwilini kuzuia kuumwa na mbu? (*Jaribu kuwashawishi wajadili)* Would you be willing to use mosquito body repellents if the above concerns are addressed*? (Prompt them to discuss)*
____
____

Q20. Je, **mngependa au mkotayari kujaribu njia nyingine za kuzuia malaria?** (*Msomee kutoka orodha ya swali la Q6).* Would you like or be willing to try additional methods to prevent malaria?
(Prompt from list in Q61) **Njia yapi?** If so which ones
____
____
